# Epigenetic Interactions between Alcohol and Cannabinergic Effects: Focus on Histone Modification and DNA Methylation

**DOI:** 10.4172/2329-6488.1000259

**Published:** 2017-03-20

**Authors:** Tiyash Parira, Alejandra Laverde, Marisela Agudelo

**Affiliations:** Department of Immunology, Herbert Wertheim College of Medicine, Florida International University, FL 33199, USA

**Keywords:** Alcohol, Cannabinoids, Epigenetics, Cannabinoid signalling, Histone modification, DNA methylation, Alcoholism, Endocannabinoids, Epigenomics, Ethanol, Histone Acetyltransferases, Histone Deacetylases, Histones, Receptor, Cannabinoid, CB1, Receptor, Cannabinoid, CB2

## Abstract

Epigenetic studies have led to a more profound understanding of the mechanisms involved in chronic conditions. In the case of alcohol addiction, according to the National Institute on Alcohol Abuse and Alcoholism, 16 million adults suffer from Alcohol Use Disorders (AUDs). Even though therapeutic interventions like behavioral therapy and medications to prevent relapse are currently available, no robust cure exists, which stems from the lack of understanding the mechanisms of action of alcohol and the lack of development of precision medicine approaches to treat AUDs. Another common group of addictive substance, cannabinoids, have been studied extensively to reveal they work through cannabinoid receptors. Therapeutic applications have been found for the cannabinoids and a deeper understanding of the endocannabinoid system has been gained over the years. Recent reports of cannabinergic mechanisms in AUDs has opened an exciting realm of research that seeks to elucidate the molecular mechanisms of alcohol-induced end organ diseases and hopefully provide insight into new therapeutic strategies for the treatment of AUDs. To date, several epigenetic mechanisms have been associated with alcohol and cannabinoids independently. Therefore, the scope of this review is to compile the most recent literature regarding alcohol and cannabinoids in terms of a possible epigenetic connection between the endocannabinoid system and alcohol effects. First, we will provide an overview of epigenetics, followed by an overview of alcohol and epigenetic mechanisms with an emphasis on histone modifications and DNA methylations. Then, we will provide an overview of cannabinoids and epigenetic mechanisms. Lastly, we will discuss evidence of interactions between alcohol and cannabinergic pathways and possible insights into the novel epigenetic mechanisms underlying alcohol-cannabinergic pathway activity. Finalizing the review will be a discussion of future directions and therapeutic applications.

## Introduction

Epigenetics, a term coined by Conrad Waddington in the early 1940’s [1] meaning “epi” or “upon” genetics, brought a whole new dimension of gene expression into light. Since then, more than 70 years later, there are over 18,000 research papers addressing this on PubMed; however, we still have a long way to go in understanding gene expression and epigenetic regulation. Epigenetics which comprises three broad umbrella systems, DNA methylation, histone modifications and non-coding RNA-mediated mechanisms, are meiotic and mitotically heritable changes that are not directly coded in the DNA sequence [2]. These epigenetic systems are associated with a group of enzymes that help carry out the modifications. In that group, some of the key enzymes are; DNA methylation mediated by DNA methyl transferases [3], histone acetylation and deacetylation mediated by histone acetyl transferases and histone deacetylases, respectively [4], and histone methylation and demethylation mediated by histone methyl transferases and histone demethylases, respectively [5]. As a general rule of thumb, an increase in DNA methylation is associated with gene silencing [6]; an increase in histone acetylation is associated with open chromatin hence increased gene transcription and vice versa for deacetylation [7]. For histone methylation, depending on the underlying histone residue, methylation/demethylation may increase or decrease the transcription of genes respectively [8]. The importance of DNA methylation and post translational histone modifications is reflected in multiple scenarios. For instance, while DNA methylation has been implicated in being involved with mammalian development and epigenetic memory [9], aberrant DNA methylation has also been reviewed to be involved in pathogenesis of atherosclerosis [10]. A recent review also discusses the role of differential DNA methylation to bring about natural human variation among populations [11]. In parallel, histone modifications are also important regulators of gene expression [12] and epigenetic alterations caused by aberrant activity of histone modifying enzymes are linked to the development of chronic diseases like cancer [13] and have even been suggested as cancer therapy targets.

To date, there is still a need to comprehend epigenetic regulation in cells to increase our understanding of mechanisms underlying several adverse chronic conditions. In one early review, it was shown that understanding the epigenetic effects involved in therapeutic drugs efficacy uncovered important epigenetic mechanisms underlying inflammatory bowel disease [14]. The article reported that changes in expression of TNF alpha and other common genes in inflammatory bowel disease are due to epigenetic factors [14]. In addition, epigenetic mechanisms underlying different forms of cancer have been extensively studied. For instance, colorectal cancer has been shown to be associated with hypermethylated DNA and gene silencing in genes related to cell cycle control, angiogenesis, and metastasis [15–17]. Hypermethylated DNA has also been implicated in prostate cancer where genes regulating hormone responses, cell cycle regulation, cell invasion, and tumor suppression have been shown to be hyper methylated [18]. In severe autoimmune diseases like systemic lupus erythematosus, multiple sclerosis, systemic sclerosis and type-1 diabetes, histone posttranslational modifications of CD4+ T cells have been heavily implicated in disease progression and pathogenesis [19].

Alcohol abuse remains a major issue in the United States and studies to elucidate the health risks and benefits of consuming alcohol, as well as the mechanisms underlying alcohol-induced end-organ effects and its addictive properties are still within the National Institute on Alcohol Abuse and Alcoholism (NIAAA) priorities. Even though several well established molecular pathways have been implicated in alcohol abuse, studying epigenetics, especially histone modifications and the genes they regulate can help identify positions of promoters and enhancers that mediate gene function and help us further understand the mechanism of action of alcohol in our body [20–22]. Therefore, this review discusses the current available literature on epigenetic mechanisms underlying alcohol abuse and cannabinoid exposure. It also brings to light connections between cannabinergic pathways and alcohol’s effects. The review aims to establish an intersecting epigenetic mechanisms, with the focus on histone modification and DNA methylation, between alcohol and cannabinergic activity. The ultimate goal is to propose directions for future studies to elucidate molecular mechanisms of alcohol abuse and hopefully outline therapeutic potential for these epigenetic targets.

### Alcohol abuse and alcohol signaling

According to NIAAA, Alcohol Use Disorders (AUDs) occur due to overuse or problem drinking of alcohol. According to NIAAA statistical report, in the year 2014, 16.3 million adults, age 18 or older had an AUD in the United States. Consumption of alcohol in levels that can be defined as problem drinking have been associated with morbidity and mortality ranging from car crashes due to Driving Under the Influence (DUI) to chronic diseases such as cancer and cardiomyopathy [23]. Adverse effects of alcohol consumption are not just limited to the primary consumer but extend to the fetus. If alcohol is being abused by the pregnant mother, the child can have severe cognitive deficits and abnormal behavior after birth [24]. Pronounced effects of alcohol exposure in human body is not limited to the liver, brain, and heart but also includes the immune system [25]. When it comes to the acute and chronic effects of alcohol, vast literature exists regarding its differential effects on human health. While observational studies have shown moderate consumption of alcohol may be protective against coronary heart disease [26], drinking in larger amounts chronically has been shown to be responsible for chronic health conditions like cancer, chronic heart diseases, and neuropsychiatric conditions [23]. In animal models, alcohol has shown to alter cholesterol, triglycerides, and very low density lipoprotein levels while also causing increased low density lipoprotein oxidation and reduced antioxidant activity [27]. Acute effects are more of a behavioral and/or mood altering quality leading to driving accidents, which depends on the amount of alcohol consumed [23]. In experimental conditions, researchers have also seen a differential effect of acute and chronic alcohol treatments in the central nervous [28] and immune systems [29,30]. A study carried out on the spleen of adolescent rats following high ethanol binge drinking showed decreased gene expression of IL-1β, IL-6, CCL2, and GABA(A) receptor α2 subunit, and increased expression of mGluR5 and 5-HT3A receptor [31]. Recently, it has also been shown that hippocampal volume is associated with lower spleen size in ethanol binge drinking adolescent rats [32]. In terms of alcohol’s effects, different pathways and receptors have been implicated in mediating these effects. For instance, some studies have shown that the acute intoxicating effects of alcohol may be linked to inhibition of certain NMDA-glutamate receptor ion channels and increasing the activity of GABAA receptor ion channels [33]. A recent review by Bell and colleagues further discusses the importance of glutamate signaling in case of alcohol abuse [34]. Of interest are the toll like receptors in liver inflammatory cells, which are activated following chronic alcohol exposure [35]. Another signaling pathway of interest in the field is the nicotinic receptor signaling pathway, which has been shown to be intricately involved in alcohol abuse and alcoholism [36]. In addition, chronic alcohol induced ROS has also been linked to ERK1/2 phosphorylation and production of TNF-α in liver immune cells [37]. Alcohol has also been shown to modulate MAP kinase signaling pathways [38,39]. Further downstream, transcription factors like NF-κB [40], STAT [41] and EGR-1 [42,43] have been shown to be modulated by alcohol exposure.

The current treatments available for heavy drinking include behavioral therapy directed towards identifying and changing behaviors that lead to heavy drinking [44] and pharmacotherapy like naltrexone, which helps reduce relapse [45]. Naltrexone has been shown to effectively reduce heavy drinking in problem drinkers [46]. However, it also has been shown to induce tolerance to its efficacy and sensitization of opioid receptors in brain [47]. Overall, even after decades of research, alcohol still does not have an effective treatment. Therefore, understanding the epigenetic basis of its mechanisms may uncover precise therapeutic targets.

### Epigenetic mechanisms of alcohol

#### Acute vs. chronic histone modifications related epigenetic effects in the liver

One of the major targets of alcohol use is damage to the liver. One of the first studies to show epigenetic modulation due to the effect of alcohol was carried out by Park et al. using rat hepatocytes treated *in vitro* with ethanol in a dose and time dependent manner. This study showed an increase in acetylation (ac) at 9^th^ lysine (K_9_) of histone (H) 3 peaking following 24 h of ethanol exposure [48]. This acetylation has an important role in the transcriptional activation of genes such as BAX [49] and FAS [50], which are important for apoptotic activity. Following this study, a similar dose and time dependent increase in H3K9ac was observed in rat hepatic stellate cells following ethanol exposure [51].

#### Acute epigenetic effects of alcohol

A modulation in H3K9ac was also observed due to acute *in-vivo* administration of ethanol to rats, in liver, lung and spleen tissues [52]. These authors also looked at methylation of the same residue and found no changes in other tissues [53]. An important factor that comes into play when studying alcohol’s effects is differentiating between whether alcohol itself or its metabolites are causing the observed effects. This was addressed, when treatment with acetate, an ethanol metabolite, also increased H3K9ac and modulated HATs in the same manner as ethanol in rat hepatocytes [54]. When it came to studying cell signaling mechanisms associated with epigenetic modulations due to alcohol, these authors also showed that inhibitors of MEK and JNK but not p38 suppressed the increase in H3 acetylation, suggesting involvement of MEK and JNK in H3K9 acetylation due to alcohol [53]. In terms of histone phosphorylation, acute ethanol treated rat hepatocytes also showed an increase in phosphorylation of H3 serine 10 and 28, which was accompanied by activation of p38 MAP kinases in the nucleus as phosphorylation was blocked by p38 MAP kinase inhibitor [54]. The same phosphorylation at H3 serine 10 and 28 was not blocked by JNK and MEK1/2 kinase inhibitors suggesting that these pathways are not mediated by H3 serine 10 and 28 phosphorylation [54].

#### Chronic epigenetic effects of alcohol

Following chronic alcohol exposure, it has been shown that there is an increase in H3K9ac in liver nuclear extracts of rats, and this effect was due to an increase in histone acetyl transferase p300 [55]. Histone acetyl transferase p300 is located at transcription sites where it binds to CBP or CREB binding protein, another histone acetyl transferase that also acts as an activator for the expression of other genes [56]. Additionally, the p300/CBP molecule interacts with a large number of proteins [56]. Therefore, an increase in p300 was considered an epigenetic phenomenon; however, more studies are required to pin-point particular genes modulated due to histone acetylation induced by alcohol [56].

#### Acute vs. chronic histone modification related epigenetic effects in the brain

Apart from the hepatic region, alcohol related epigenetic effects have also been studied in the brain. An increase in acetylation in histone H3 and H4, CREB Binding Protein (CBP) and a corresponding decrease in HDAC activity was seen in rat amygdala due to acute alcohol exposure and these effects were reduced when treated with the HDAC inhibitor, trichostatin A during ethanol withdrawal [57]. A study in a rodent model showed that chronic intermittent alcohol exposure increased H3K9ac and H4K12ac in the frontal cortical region of young rats; however, the same alteration was not observed after adult exposure [58]. An increase in H3K4 methylation due to alcohol exposure has also been seen in alcoholic brains [59] and particularly in the hippocampus [60]. This increase in H3K4 trimethylation along with activation of several other genes was found to be involved in transcription co-repressor complexes [59]. These authors also confirmed, from other literature, an upregulation of Methyl-CpG binding domain (MBD) in the alcoholic brain [59,60]. This is an important transcription co-repressor complex protein necessary for pairing HDAC activity to chromatin remodeling [59]. A summary of these studies is listed in table 1.

In the context of histone deacetylation, studies from our laboratory, have demonstrated acute alcohol increases HDAC 1 and 3 gene expression and HDAC activity in CNS cells along with an increase in serotonin receptor 5-HT3 activity [61]. Moreover, additional studies from our group have demonstrated acute alcohol treatment of SK-N-MC increases HDAC2 while partial inhibition of HDAC2 using non-specific HDAC inhibitor, TSA, showed neuroprotective effects [62].

Acute vs. chronic histone modification related epigenetic effects in the immune system and other peripheral organs: Recently, even in the immune system, alcohol was shown to increase class I HDACs *in-vitro* in primary human Monocyte-Derived Dendritic Cells (MDDCs) treated acutely with alcohol and also in MDDCs from alcohol users [63]. Also consistent with our findings, Lopez-Moreno and colleagues demonstrated HDACs 1–11 are differentially modulated in the periphery following acute binge alcohol and repeated chronic binge alcohol administration of both rats and humans [64].

Apart from studies in liver, brain, and immune system, some studies done in other peripheral organs also show the epigenetic effects of alcohol. A recent study showed chronic alcohol reduced methylation at H3K4 and increased H3K27 trimethylation in a pancreatic cell line, modulating its ability to uptake ascorbic acid [65]. Alcohol exposure during pregnancy has been associated with congenital heart disease [66]. Research has shown that alcohol increased acetylation at H3K9 in heart progenitor cells and consequently increased expression of heart development related genes [67]. Additionally, the same group showed that curcumin, a HAT inhibitor, could reduce the hyper-acetylation due to alcohol and correct the expression of development related genes in cardiac progenitor cells [68]. In summary, when it comes to histone modifications, regardless of the specific region or tissue, H3K9ac, H3K4me, H3K27me and H4K12ac emerge as important epigenetic downstream effects following alcohol abuse or exposure. Additionally, HDACs and HATs seem to play an essential role in these modifications.

#### Alcohol and DNA methylation

Besides histone modifications, alcohol has also been widely associated with modulation in DNA methylation. DNA methylation, which is mediated by DNMTs, transfer a methyl group from S-adenosyl methionine (SAM) to 5-methyl cytosine and produce S-adenosyl homocysteine, which is then broken down by SAH Hydrolase (SAHH) to form homocysteine [69,70]. Studies have shown that folates and other B vitamins are crucial for synthesis of SAM [71]. Alcohol has been shown to modulate vitamin B and folate synthesis in the body [71,72]. Thus, appears that alcohol can indirectly modulate DNA methylation through the diet. Besides dietary influences, metabolites of alcohol like acetaldehyde have been shown to modulate DNA methylation by inhibiting DNA methyl transferases [73].

#### Acute effects of alcohol on DNA methylation

It was recently shown that binge ethanol exposure during adolescence causes altered genome-wide DNA methylation patterns in the hypothalamus of offspring who have never been exposed to alcohol [74]. A study which looked at epigenetics of neural stem cell differentiation under the effect of binge like alcohol exposure found that alcohol hinders DNA methylation affecting the functionality of certain important neural stem cell genes and impeding stem cell differentiation in the process [75]. Among other critical genes that were affected, some physiologically significant genes are insulin-like growth factor 1 (Igf1), in cases of eye development, lens intrinsic membrane protein 2 (Lim 2); the epigenetic mark Smarca2 (SWI/SNF related, matrix associated, actin dependent regulator of chromatin, subfamily a, member 2); and developmental disorder DiGeorge syndrome critical region gene 2 (Dgcr2) [75].

#### Chronic effects of alcohol on DNA methylation

**Figure 1:**
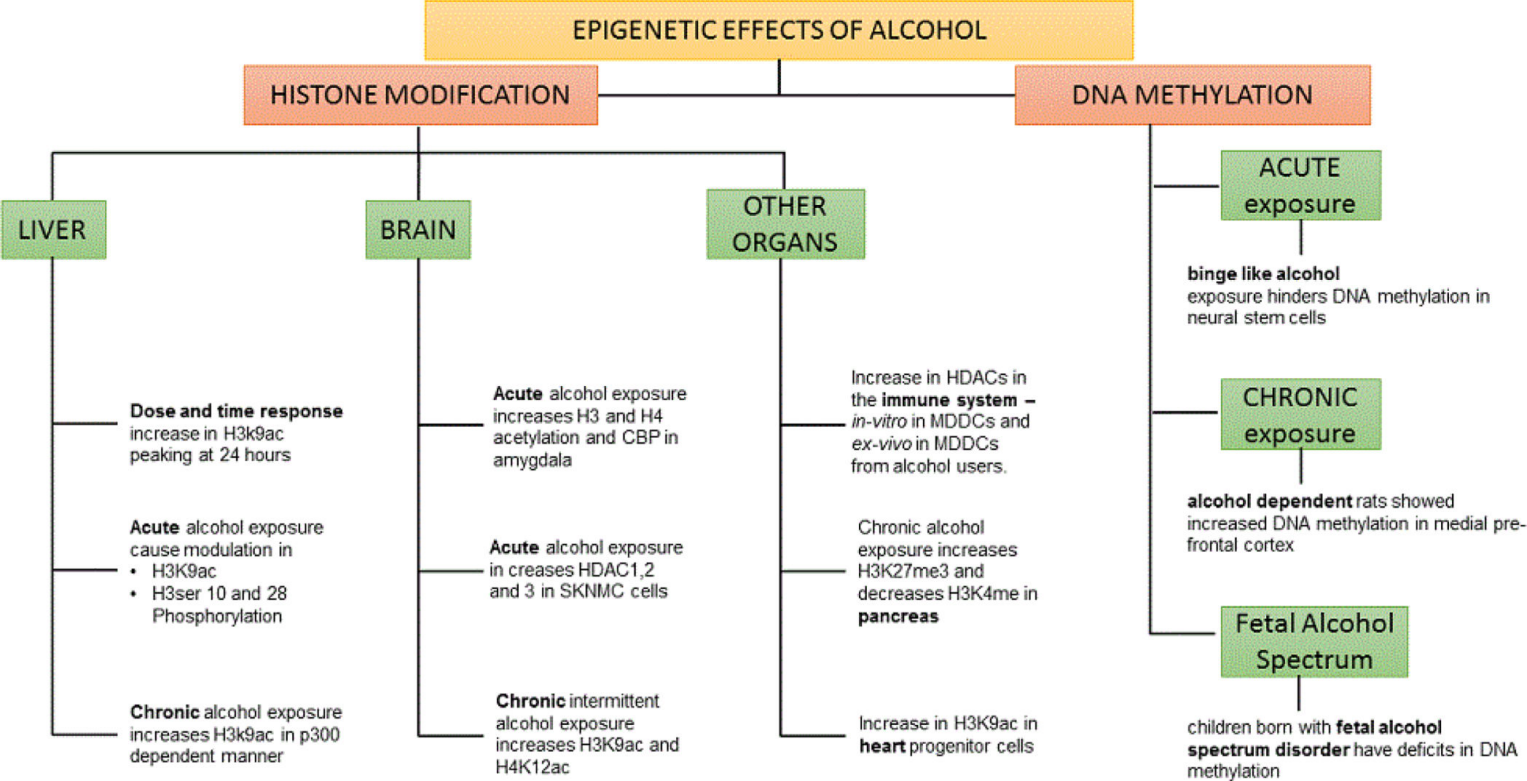
Schematic flow chart of literature summarizing epigenetic effects of alcohol.

A recent study that looked at a possible role of DNA methylation and its association with alcohol dependence found that alcohol dependent rats showed increased DNA methylation in medial pre-frontal cortex as well as decreased expression of proteins involved in synaptic neurotransmitter release [76]. Moreover, addition of a DNA methyltransferase inhibitor rescued the increased drinking behavior post abstinence suggesting prospective therapeutic potential for DNA methyltransferase inhibitors [76]. Another study showed a similar effect of alcohol in neuronal plasticity through decreased DNA methylation levels at the tissue plasminogen activator (tpa) promoter [77]. A recent study done in children with fetal alcohol spectrum disorder looked at the DNA methylation patterns from buccal swabs using arrays [78]. The study, even though it included only 6 patients and 6 controls, confirmed other results showing that children born with fetal alcohol spectrum disorder have deficits in DNA methylation [78]. Gene studies underlying these unique CpG methylation patterns found that these gene clusters were from the protocadherin gene family which mediates complex cell to cell interactions and glutamatergic pathways [78]. These, in turn, are involved in synaptic plasticity, learning, and memory [78]. In summary, with respect to alcohol’s effects on DNA methylation, studies show alcohol is able to differentially modulate DNA methylation affecting synaptic plasticity and cell development by modulating functionality of gene clusters related to the glutamatergic pathways [78] and specific genes like Igf1 and LIM 2 amongst other genes [75]. These studies have been summarized in a schematic flow chart in Figure 1 [79].

Even though, micro RNA based epigenetic modulations have not been discussed in this review, it is of importance to mention that immense number of studies have shown specific micro RNA are modulated due to alcohol abuse as reviewed by Szabo and Bala [80]. Specifically, microRNA-122 is emerging to be a common marker for multiple liver diseases including alcoholic liver disease [80,81]. Further, certain micro RNA based epigenetic modulations due to alcohol have also shown to be associated with the GABAA receptor [82].

Overall, according to the literature reviewed on alcohol and epigenetics, epigenetic mechanisms, including both DNA methylation and histone modifications have been implicated in both the acute and chronic effects of alcohol in different organs such as the brain, liver, and immune system. However, most of these are in animal models and there continues to be a need to elucidate alcohol’s epigenetic effects in humans. Our current laboratory efforts are focused on understanding epigenetic modifications induced by alcohol in human peripheral immune cells, with the hope of establishing certain epigenetic markers and drug targets for AUDs.

### Cannabinoid abuse and endocannabinoid signaling

According to the National Institute on Drug Abuse (NIDA), marijuana or cannabis is currently the most common illicit drug abused in the United States [83]. While the immediate effects of cannabis use include altered senses, thinking ability and movement, there are adverse effects following long term use such as breathing problems, increased heart rate and problems with child development throughout and after pregnancy [84]. Apart from the major psychoactive cannabinoid, Δ^9^-tetrahydrocannabinol (THC) [85], there are other cannabinoid compounds that have medicinal properties. Cannabidiol is one such compound which has been shown to have potential therapeutic applications [86]. THC also has been associated with therapeutic potential against simian immunodeficiency virus infection in rhesus macaques [87].

Endocannabinoid signaling relies on G-protein-coupled receptors (GPCRs), which are the main receptors responsible for the action of cannabinoids [88]. CB_1_ receptor is primarily found in the brain, with lower levels in the periphery. A second cannabinoid receptor, CB_2_, is primarily found in immune cells and cells of the hematopoietic system [89] and appears to be scarcely present in the brain [90] and pancreas [91]. Another non CB_1_/CB_2_ receptor, receptive to cannabinoids is GPR55, which is found in the human brain [92], liver and peripheral immune cells as well as the rat intestines and spleen [93,94]. It is also present in the gastro-intestinal tract [95]. Researchers also showed that GPR55 is targeted by a number of cannabinoids and, interestingly, it is activated by THC with the same potency but with greater efficacy than the CB_1_ receptor [96].

As recently reviewed, when CB1 and CB2 receptors get activated they modulate adenylate cyclase and correspondingly production of the secondary messenger cAMP further affecting the protein kinase A pathway [97]. CB_1_ and CB_2_ receptors also activate MAP kinases, ERK kinases, and nuclear factors like NF-κB downstream signaling pathways [97]. When studying signaling mechanisms of the non-CB1/CB2 cannabinoid receptor, GPR55, researchers have been in dilemma as specific endogenous ligands for GPR55 are still under scrutiny, and several cell-signaling mechanisms have been associated with it. Studies have shown GPR55 is coupled to Gα13, but not Gαs, Gαi, or Gαq [96]. Other researchers have shown that GPR55 activation altered several signaling mechanisms, elevated calcium levels, activated MAP kinases ERK1/2 via Rho pathways, and in turn, activating transcription factors CREB and NF-κB [98].

### Epigenetic mechanisms of cannabinoids

Understanding epigenetic mechanisms by which cannabinoids bring about their effects may lead to a deeper understanding of their molecular mechanism of action. Although there is limited literature on the epigenetic mechanisms regulating cannabinoid signaling, one of the pioneering reports showed, in an *in-vitro* study, anandamide reduced differentiation of keratinocytes by inducing DNA methylation of the CB_1_ receptor [99]. Paradisi and colleagues described, for the first time, CB_1_ receptor mediated DNA methyltransferase activity, which was proposed to be involved with many more functions other than just differentiation of keratinocytes [99]. More recently, studies have been carried out to look into histone modifications due to cannabinoid signaling. THC modulated multiple histone modification sites like H3K4me3, H3K9me3, H3K27me3, and H3K36me3 in differentiating mouse lymph node cells showing histone modifications are associated with THC-mediated alterations in antigen-specific T cell responses [100]. Additional cutting edge reports have emerged which connects alterations in DNA methylation status to the effects of cannabinoids. For instance, a study reported that parental exposure to THC altered DNA methylation status of genes related to synaptic plasticity in rat nucleus accumbens [101]. Another recent study, done in mice, showed that THC administration increased methylation at the promoter region of DNA methyltransferases 3A and 3B in myeloid derived immune suppressor cells, and correspondingly reduced expression of the same DNA methyltransferases [102]. Additionally, methylation at the promoter regions of Arg1 and STAT3 were decreased by THC which led to further increases in levels of Arg1 and STAT3 expression. Arg1, which can metabolize L-arginine, suppresses T-cell function [103] while increasing activation and function of these immunosuppressive cells [104]. Cannabinoid signaling has also been shown to be associated with modulation in certain microRNA based epigenetic mechanisms [105,106], however for the scope of this review they have not been discussed in detail. To summarize, these reports on epigenetic effects of cannabinoids reveal the ability of cannabinoids to modify neuronal and immune cell functionality either via histone modifications like H3 lysine methylations or by altering DNA methylation. These reports along with a recent review of the literature are described in Table 1.

### Alcohol and cannabinergic pathway associations

Alcohol and its association with the endocannabinoid system, which is involved in reward signaling mechanism of the brain, has been studied quite extensively with cannabinoids and its receptors being explored as pharmacological drug targets for AUDs [107]. The cannabinoid system appears to be important in regulating addiction by influencing retrograde signaling to suppress neurotransmitter release [107]. This synaptic activity plays a role in the mechanism underlying addiction to substances of abuse including alcohol [107]. Several studies have reported the ability of cannabinoid receptor agonists/ antagonists to modulate ethanol self-administration in rodents. For instance, chronic ethanol exposure has been shown to down-regulate CB_1_ receptor function, which may be due to constant stimulation by the endogenous cannabinoids, arachidonylethanolamide and 2-arachidonylglycerol, with this activity also modulated by chronic ethanol exposure [108]. The CB_1_ cannabinoid receptor and the nuclear peroxisome proliferator-activated receptor PPARα were found to be lower in spleens of intermittently alcohol-exposed adolescent rats compared to control rats [109]. Conversely, CB_2_ expression was higher, indicating that alcohol exposure even during adolescence was enough to cause difference in splenic expression of cannabinoid receptors [109]. Another recent report in which CB_2_ receptor knockout or apoptosis related gene ATG5 knockout mice were exposed to chronic ethanol followed by binge ethanol feeding showed that CB_2_ receptor activation in liver macrophages protects mice from alcohol-induced steatosis by blocking hepatic inflammation through an autophagy-dependent pathway [110]. In human studies, cannabinoid signaling has been associated with alcohol exposure as recently reviewed in 2016 [111]. Exposure to ethanol increases the activity of the endocannabinoid system in the brain, leading to decreased CB1 binding and uncoupling that is directly associated with consumption of ethanol [111]. Furthermore, a positron emission tomography imaging study revealed acute alcohol exposure in humans increased cerebral CB_1_ binding while chronic alcohol exposure caused a decrease in CB_1_ binding that is maintained even after abstinence for at least a month [112]. The enhancement of CB1 receptor levels is being explored as a therapeutic mediator of the negative affective state caused by alcohol withdrawals, which is critical for the treatment of AUDs [112].

Apart from the brain and liver, in a recent review comparing the differential effects of alcohol and cannabinoids on the immune system, marijuana abusers were shown to have reduced secretion of inflammatory cytokines from peripheral dendritic cells compared to alcohol users [113]. Additionally, previous reports from our laboratory showed increased expression of cannabinoid receptors CB_2_ and GPR55 in MDDCs from alcohol users, which was further validated *in-vitro* in primary human MDDCs treated with alcohol [94]. All of these provide support for the existence of a connection between endocannabinergic signaling pathways and alcohol abuse.

### Epigenetic mechanisms underlying alcohol-cannabinergic pathway associations

Regarding studies examining epigenetic mechanisms underlying alcohol and cannabinoid activity, a recent study reported post-natal alcohol exposure induced neonatal neurodegeneration by enhancing CB1 exon1 activity through upregulated histone H4K8 acetylation along with downregulated H3K9 methylation [114]. This appears to have caused upregulation of CB1 function leading to neurobehavioral abnormalities in adult mice [114]. The enhancement of CB1R activity is able to suppress the action of neurotransmitters and prevent recruitment of new synapses leading to memory disruption [114]. Another study examined DNA methylation regarding alcohol and cannabinoid activity association [115]. The report showed, in a mouse model of fetal alcohol spectrum disorder, perinatal alcohol exposure of mice caused activation of caspase-3 in neonatal mouse brain [115]. Further, perinatal alcohol exposure impaired DNA methylation through downregulation of DNA methyl transferases DNMT1 and DNMT3A in the neonatal brain and such losses were absent in CB1 receptor null mice [115]. Additionally, blockade of CB1 receptor prior to ethanol treatment in wild type mice also rescued the loss of DNA methylation [115]. These reports suggest the potential of epigenetic overlap between alcohol and cannabinergic activity.

**Figure 2:**
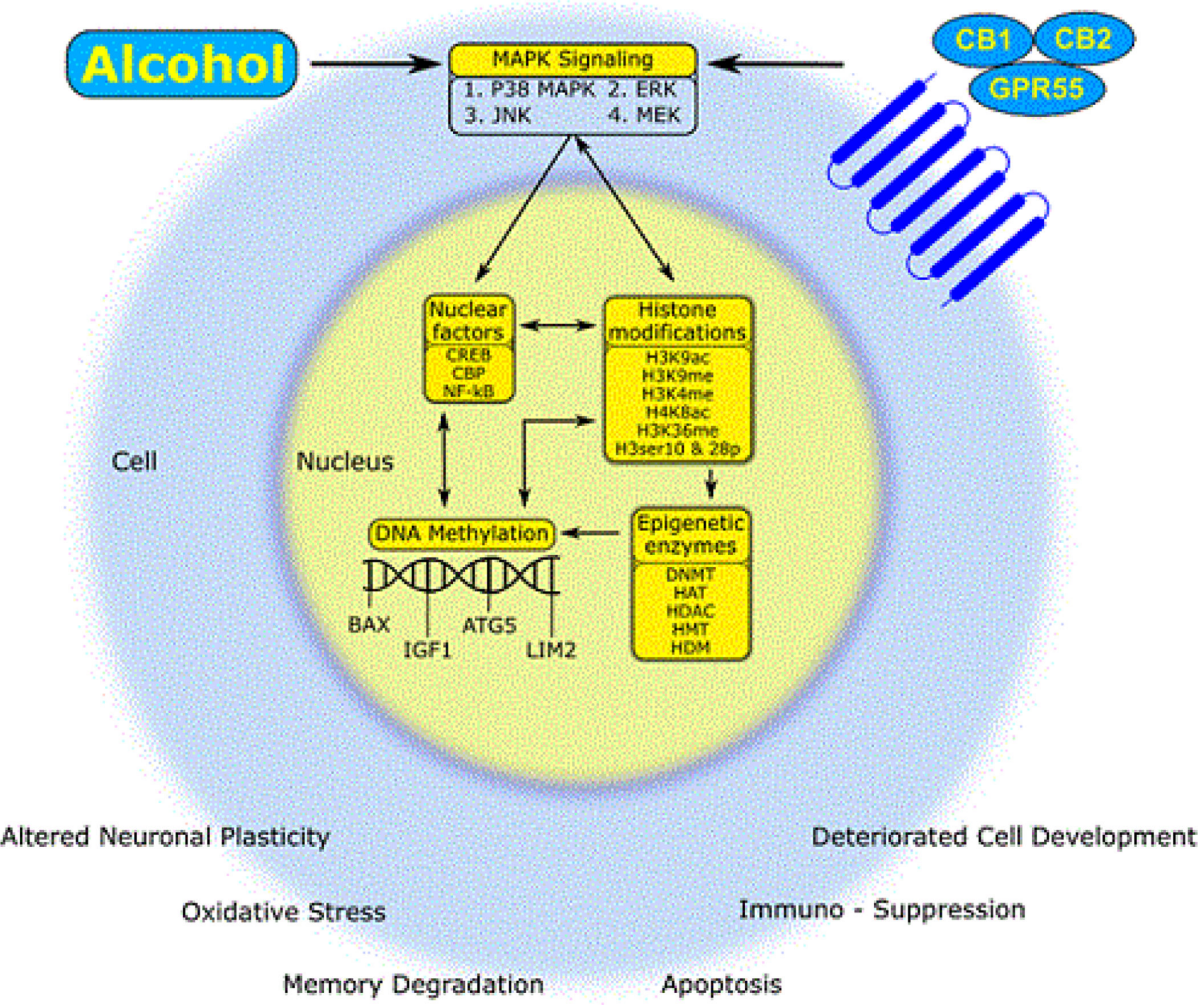
Schematic summary of the epigenetic modifications induced by alcohol and/or cannabinoids. Alcohol and cannabinoids activate the MAP kinase signaling pathways, subsequently activating nuclear factors like CREB and NF-κB, histone modifications, and DNA methylation, which are mediated by the epigenetic enzymes, finally leading to altered gene expression and cell functionality through apoptosis, oxidative stress, plasticity or immunomodulation.

Even though there is a dearth of studies looking into the epigenetic mechanisms behind alcohol and cannabinoid activity, certain inferences can be drawn from the studies reviewed herein. Histone modification sites like H3K9, H3K4 and H4K8 have been commonly implicated in these studies. DNA methylation also seems to be significantly influenced by both alcohol and cannabinoids. It is important to isolate these epigenetic effects and understand their functionality. Interestingly, in 2004, the first histone Lysine Specific Demethylase 1 (LSD1) was identified by Dr. Shi Yang’s group [116]. Further, LSD1 was found to demethylate repressive histone marks like H3K4me1/2 and H3K9me1/2 to promote androgen-receptor-dependent transcription [117]. Another interesting approach to look deeper into an epigenetic connection between alcohol and cannabinoid activity would be to do associative studies between histone modification and DNA methylation. It is known that DNA methylation may act in synchrony with histone modifications leading to tighter regulation of gene expression. For instance, they may act together to form a transcriptionally inactive chromatin state. This would be done by binding proteins such as MeCP2, which subsequently binds to methylated DNA bringing HDAC enzymes in contact with methylated promoters resulting in gene silencing [118,119]. Additionally, DNA methyltransferases can recruit HDAC enzymes directly to further mediate gene silencing [120,121]. Similarities between molecular mechanisms underlying alcohol induced epigenetic regulation such as p38 MAP kinases, JNK, MEK kinases in turn, activating nuclear factors like CREB and NF-κB via cannabinergic signaling suggest possible epigenetic overlap between these two drugs of abuse. A schematic diagram Figure 2, represents the epigenetic mechanisms of alcohol and cannabinoid activity and possible hypothetical overlaps between the two at the epigenetic level. Thus, drugs which can target these master regulators can serve as potential therapeutic targets to treat AUDs and other substance abuse [122,123].

## Conclusion and Future Directions

The adverse effects of alcohol and cannabinoid abuse are widely known; however, with the legalization of marijuana in some states and availability of medical marijuana, the potential of endogenous cannabinoids and their receptors to be of therapeutic significance is immense. From the review above, we can see a vast existing literature showing the connection between effects of alcohol and cannabinoid activity, such that cannabinoid receptor agonists and/or antagonists may be able to reverse alcohol’s deleterious effects or reduce alcohol self-administration. Understanding epigenetics may help us ultimately understand the connection between alcohol and endocannabinoid signaling mechanisms. In summary, as shown by the schematic representation in Figure 2, cannabinoids and alcohol activate similar cell signaling events that can trigger various common nuclear factors, histone modifications, and DNA methylation resulting in functional effects like apoptosis and synaptic plasticity.

**Figure 3:**
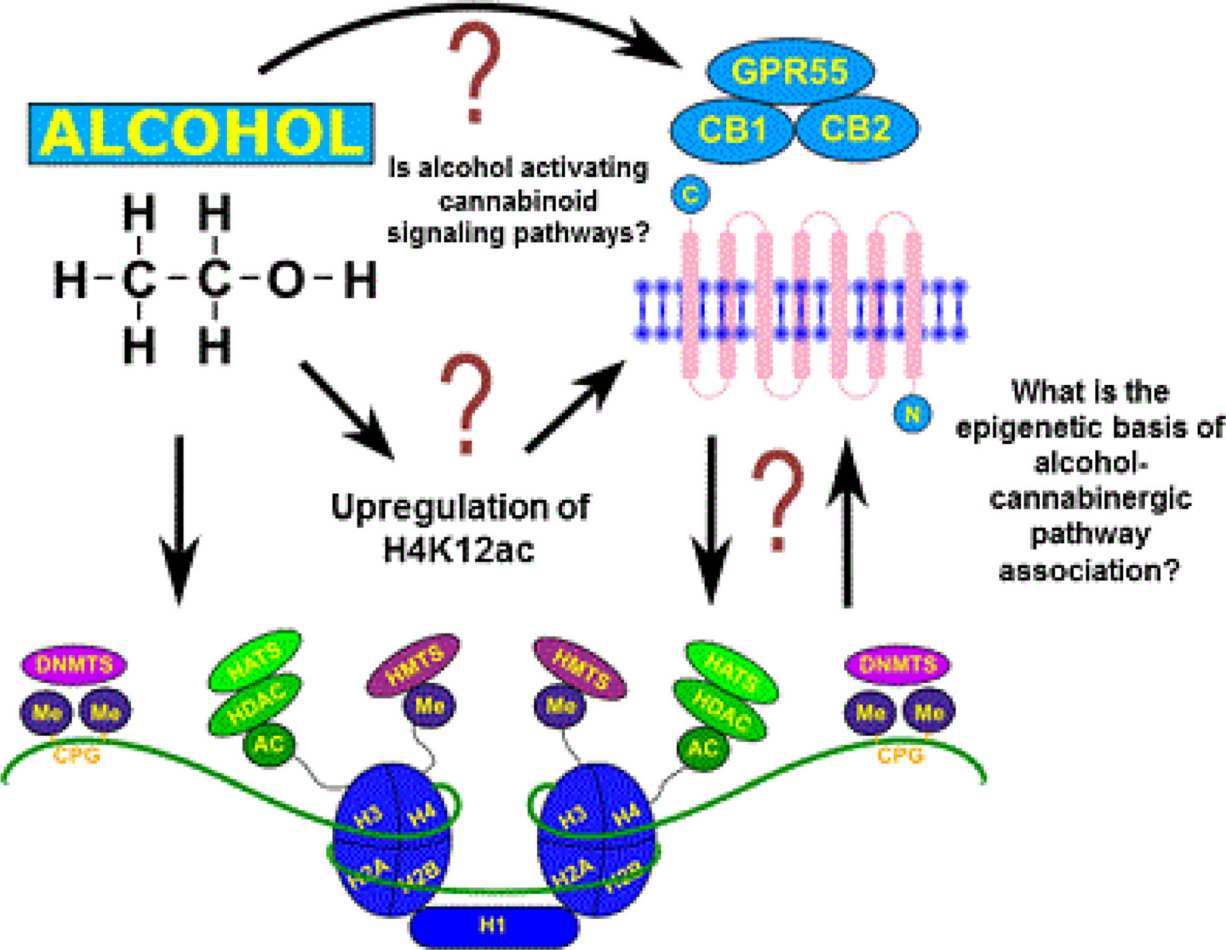
Posited future direction to elucidate the epigenetic basis of alcohol-cannabinergic pathway associations. We have demonstrated that alcohol induces cannabinoid receptors activity in monocyte-derived dendritic cells. In addition, our group has preliminary evidence on the ability of chronic alcohol exposure to induce H4K12 acetylation. However, whether this increase in acetylation is in turn associated with the regulation of cannabinoid receptors and cannabinoid signaling pathways need further investigation. Therefore, the epigenetic basis of alcohol-cannabinergic pathway association’s need continued examination.

Considering the similarities in effects that exist between the two at the cellular level, there may be possible overlap between these two pathways that can reveal therapeutic targets. As shown in Figure 3, the epigenetic bases of alcohol and cannabinergic pathway activity are still understudied. Therefore, studies using HDAC, HAT or DNA methylation inhibitors to analyze cannabinoid gene and/ or promoter activity by ChIP-sequencing after acute and chronic alcohol exposure may elucidate epigenetic mechanisms underlying AUDs and their detrimental effects on the human body.

After reviewing the literature, there are many questions that still remain to be elucidated (Figure 3) including whether alcohol’s effects involving cannabinergic receptors bring about its adverse effects and whether epigenetics are responsible for these observed effects. Therefore, studies to analyze gene promoter occupancy and the presence of cannabinoid receptors during alcohol-modulating epigenetic events are necessary. In addition, other studies analyzing epigenetic modifications at cannabinergic receptor genes due to alcohol exposure will be beneficial in advancing the field. However, it is crucial to take into consideration that epigenetic modulations are differentially regulated depending on the levels of alcohol exposure and the experimental model being used, as we saw in fetal alcohol exposure models discussed above [114,115]. In summary, in an effort to elucidate the epigenetic overlaps between the effects of alcohol and cannabinoid activity, we provided herein a review of the available literature and suggestions for future directions, which may elucidate epigenetic mechanisms involved in alcohol abuse and the development of pharmacotherapies to treat this disorder.

## Figures and Tables

**Table 1:** Epigenetic modifications induced by alcohol and/or cannabinoids. A comprehensive table showing up to date contributions from the literature. The first column shows the group that carried out the research study, second column indicates the epigenetic modifications associated with alcohol and/or cannabinoids, and the last column indicates the experimental model and region used in the study.

Alcohol		
Group	Epigenetic Modifications	Experimental Model/Region of Study
Park et al. [49]	Increase in H3K9ac in hepatocytes	Acute alcohol exposure in rat hepatocytes
Kim and Shukla [51]	Increase in H3K9ac in stellate cells	Rat hepatic stellate cells
Park et al. [53]	Increase in association of class I alcohol dehydrogenase (ADH I) gene with H3K9ac, increase in HAT activity	Acute alcohol treatment in rat hepatocytes
Kim and Shukla [52]	Increase in H3K9ac in liver, lung spleen tissues	Acute *in-vivo* alcohol administration to rats
Lee and Shukla [54]	Increase in phosphorylation of H3 serine 10 and 28, along with nuclear activation of p38 MAP kinase	Acute ethanol treatment in rat hepatocytes
Pandey and Ugale [57]	Increase in H3 and H4 acetylation, decrease in HDAC activity at acute levels	Acute alcohol exposure in rats, study carried out in the amygdala region
Pascual [58]	Increase in H3K9ac and H4K12ac	Chronic intermittent ethanol administration in young rats, study carried out in frontal cortex region
Zhou [60]	Genome wide alteration in H3K4me3 in hippocampus	Human post mortem brain tissue from alcohol and cocaine users
Ponomarev [59]	Increase in H3K4me3 and associated DNA hypo methylation	Human autopsy brain samples from alcoholics and non-alcoholics
Agudelo [61]	Increase in HDAC 1 and 3 and HDAC activity along with increase in 5-HT3	Acute alcohol treatment in CNS cells
LópezMoreno [62]	Differential modulation in HDACs 1–11	Acute and chronic alcohol exposure in rats and human periphery.
Agudelo [63]	Increase in class I HDACs gene expression and differential modulation in HDAC protein expression	Acute alcohol treatment in primary monocyte derived dendritic cells *in-vitro* and from alcohol users
Asimes [74]	Alteration genome wide DNA methylation in alcohol-naïve offspring	Binge ethanol exposure in adolescent rats, study carried out in hypothalamus of offspring
**Cannabinoids**		
**Group**	**Epigenetic Modifications**	**Experimental Model/Region of Study**
Khare [124]	Increase in HDAC3 expression causing dysregulation in genes for placental development	THC exposure in Human trophoblast cell line BeWo
Paradisi [99]	Modulation in DNA methylation at CB1 receptor	Anandamide exposure to keratinocytes
Tomasiewicz [125]	Decrease in H3K9me2 and me3 levels associated with Penk gene mRNA levels	THC exposure to adolescent rats and studies carried out in adult rat nucleus accumbens
DiNieri [126]	Increase in H3K9me2 and decrease in H3k4me3 at the Drd2 gene locus in offspring	in utero THC exposure to rats followed by studies in adult rat nucleus accumbens
Yang [100]	Modulation in multiple histone modification sites like H3K4me3, H3K9me3, H3K27me3, and H3K36me3	THC exposure in differentiating mouse lymph node cells
**Alcohol-Cannabinergic Pathway**		
**Group**	**Epigenetic Modifications**	**Experimental Model/Region of Study**
Subbanna [114]	Increase in H4K8ac, decrease in H3K9me by enhancing CB1R exon	Postnatal alcohol exposure in rats, studies carried out in neonatal brain.
Nagre [115]	Downregulation in DNMT1 and DNMT3A and impairment of DNA methylation, while CB1R knockout mice does not show the same deficits	Postnatal alcohol exposure and studies carried out in neonatal brain.
